# Epigenetic silencing directs expression heterogeneity of stably integrated multi-transcript unit genetic circuits

**DOI:** 10.1038/s41598-021-81975-1

**Published:** 2021-01-28

**Authors:** Jan Zimak, Zachary W. Wagoner, Nellie Nelson, Brooke Waechtler, Hana Schlosser, Morgan Kopecky, Jie Wu, Weian Zhao

**Affiliations:** 1 Sue and Bill Gross Stem Cell Research Center, University of California, Irvine, Irvine, CA 92697 USA; 2 Department of Pharmaceutical Sciences, University of California, Irvine, Irvine, CA 92697 USA; 3 Chao Family Comprehensive Cancer Center, University of California, Irvine, Irvine, CA 92697 USA; 4 Edwards Life Sciences Center for Advanced Cardiovascular Technology, University of California, Irvine, Irvine, CA 92697 USA; 5 Department of Biomedical Engineering, University of California, Irvine, Irvine, CA 92697 USA; 6 Department of Biological Chemistry, University of California, Irvine, Irvine, CA 92697 USA

**Keywords:** Gene therapy, Genetic engineering

## Abstract

We report that epigenetic silencing causes the loss of function of multi-transcript unit constructs that are integrated using CRISPR-Cas9. Using a modular two color reporter system flanked by selection markers, we demonstrate that expression heterogeneity does not correlate with sequence alteration but instead correlates with chromosomal accessibility. We partially reverse this epigenetic silencing via small-molecule inhibitors of methylation and histone deacetylation. We then correlate each heterogeneously-expressing phenotype with its expected epigenetic state by employing ATAC-seq. The stability of each expression phenotype is reinforced by selective pressure, which indicates that ongoing epigenetic remodeling can occur for over one month after integration. Collectively, our data suggests that epigenetic silencing limits the utility of multi-transcript unit constructs that are integrated via double-strand repair pathways. Our research implies that mammalian synthetic biologists should consider localized epigenetic outcomes when designing complex genetic circuits.

## Introduction

The ability to engineer the genomes of mammalian cells with exogenous genetic circuits is central to the goal and applications of mammalian synthetic biology^1^. Many upcoming therapeutic applications of synthetic biology employ designer cells engineered with genetic circuits^2–4^. Genetic circuits in synthetic biology are generally composed of multiple transcription units (TUs). Recently, standardized modular DNA assembly methods have been used to construct multi-TU vectors^5^ that enable multiple different genes to be expressed under the control of multiple different promoters. While these methods have been extensively used in prokaryotes and simple eukaryotes^6,7^, multi-gene circuits have been used in transient expression^8^, genomically-integrated modular gene circuits have not been widely adopted within the mammalian synthetic biology community since their initial development. Duportet et al.^9^ developed a site-specific recombinase-mediated method of integrating large modular multi-TU genetic circuits into mammalian cells. Each TU is separated by chromatin insulators designed to block adjacent TUs from affecting each other as well as maintaining the whole genetic circuit in a euchromatin state^10,11^. Such circuits can be integrated into monoclonal chassis cell lines, termed “landing pads,” using the Bxb1 recombinase at high efficiency. Landing pad cell lines first require the integration of recombinase sites at only one chromosomal location, achieved by causing double-stranded breaks using a targeted nuclease (such as zinc fingers or CRISPR/Cas9) and relying on the cells’ endogenous double-stranded break repair pathways to integrate the vector. Thus, two sequential steps are required to engineer new cell lines. Given the progress in improving CRISPR/Cas9 editing efficiency^12–14^, these modular systems could be integrated in one step. However, Duportet et al.^9^ showed that modular constructs integrated in this manner heterogeneously express each TU, rendering any resulting genetic circuits non functional. This expression heterogeneity could be caused by sequence alterations to the constructs during integration^9^, chromatin changes imparted during integration^15^, locus-mediated inhibition of expression^16^, or insufficient chromatin insulation^17^. While silencing of transgene expression in mammalian cells has been extensively studied^18^, it has not been studied in the context of large multi-gene constructs.

The function of stably integrated genetic circuits breaks down when any circuit element is silenced and thus these systems are extremely sensitive to variations in gene expression or time-dependent silencing^18^. Transgene silencing and variations in expression have been linked to epigenetic mechanisms, such as DNA methylation, histone deacetylation and chromatin accessibility at the site of integration^18^. While regulatory elements such as chromatin insulators have been used to activally remodel local chromatin interactions^19^ and passively block heterochromatin spread^20^. These regulatory elements have been improved in recent years, by targeting sequences from ubiquitous chromatin opening elements (UCOE)^21,22^, as well as discovery of novel CTCF-binding motifs with 10 times the insulating capacity of the canonical cHS4 chromatin insulator^17^.

Here we report an approach to identify the causes of expression heterogeneity in multi-TU genetic circuits integrated using endogenous DNA-repair pathways. To enable this, we developed a modular multipart assembly method utilizing a hierarchical cloning method. Using this method, we constructed a four TU vector comprised of two fluorescent reporters flanked by two independent selection markers each under the control of separate constitutive promoters. After integration of our two color reporters, we isolated each expression phenotype and performed genetic and epigenetic analyses to identify possible mechanisms. We also show that position-dependent use of selection markers controls which expression phenotype dominates and that repetitive screening reinforces each expression phenotype, indicating that continued epigenetic remodeling occurs for over one month after integration. Then we examined how expression phenotype matches the local epigenotype using a genome-wide chromosomal accessibility assay (ATAC-seq). Lastly, we show that phenotypes are partially reversed using small-molecule inhibitors of methylation and histone deacetylation. Our results indicate that widespread application of multi-TU genetic circuits will likely require strategies that can mitigate local epigenetic effects of integrated genetic circuits.

## Results

### Expression heterogeneity stabilizes into distinct expression phenotypes

**Figure 1:**
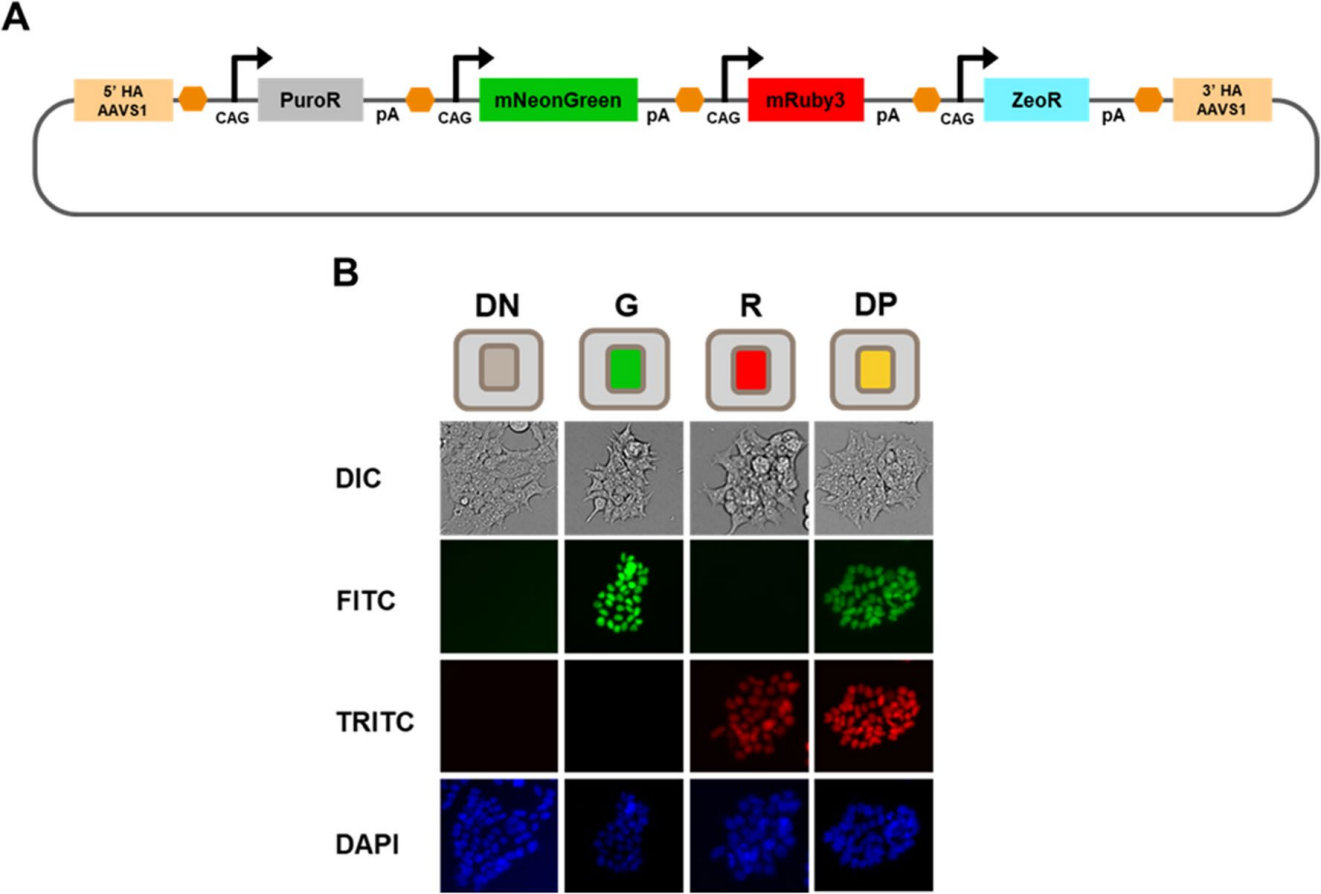
Heterogeneous expression of multi-gene construct. (**A**) Schematic showing our multi-gene construct design in which expression of four genes (PuromycinR, mNeonGreen-NLS, mRuby3-NLS, and ZeocinR) is constitutively driven by independent CAG promoters. Genes are flanked by CTCF insulators (orange) and WPRE-bGHpolyA (pA) sequences. The multi-gene transcripts are then flanked by 5′ and 3′ homology arms (HA) directing insertion into the AAVS1 loci. (**B**) Fluorescent imaging representative of the four stable and distinct phenotypic populations observed after undergoing multiple rounds of puromycin selection at 14 days post-transfection. *DN* Double negative, *G* mNeonGreen-NLS only, *R* mRuby3-NLS only, *DP* Double Positive.

We set out to build a model system to better understand expression heterogeneity in a multi-TU system. This was constructed using a hierarchical Gibson assembly method similar to previous methods^23^. Our model system contained four TUs in the following order: (1) PuromycinR, (2) nuclear localized-mNeonGreen, (3) nuclear localized mRuby3, and (4) ZeocinR (Fig. 1A). Using this system, we can functionally test for the expression of all four transcripts using either resistance to selection or the visual presence of fluorescent reporters.

**Figure 2:**
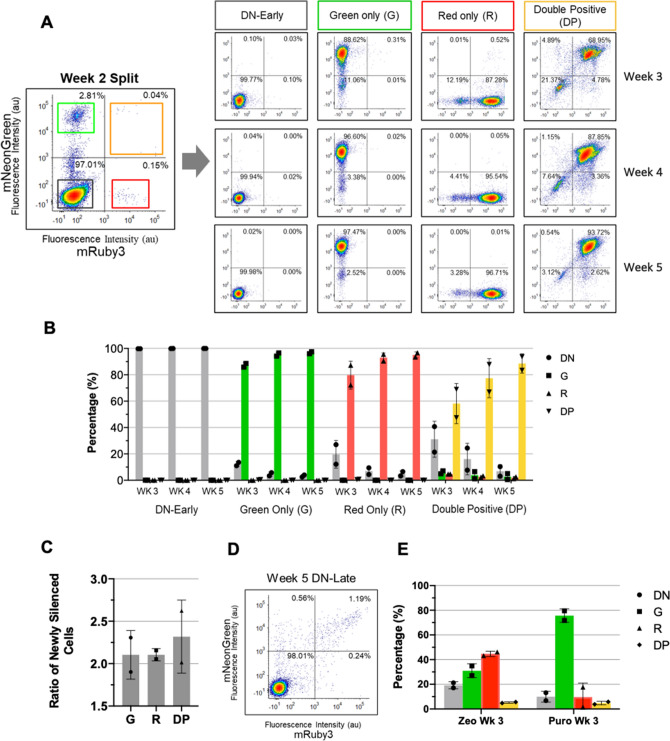
Characterization of expression phenotype dynamics using screening and selective markers. (**A**) Transfected HEK293T cells were selected with puromycin for two weeks following transfection with our model system construct prior to sorting via FACS into four expression phenotypes (DN, G, R, and DP). Each phenotypic state was further sorted each following week until 5 weeks where cells were gated and isolated for their respective phenotype (Ex: Green only cells at week 3 were sorted for the 88.62% mNeonGreen- population and replated for sorting the following week). (**B**) Summary of temporal data for the DN, G, R, and DP populations, generated from biological duplicates, over 5 weeks of FACS sorting. Fluorescent percentage refers to percent of cells in specific expression phenotype. (**C**) Average ratios of newly silenced DN cells (from week 3 to week 4 and from week 4 to week 5). (**D**) Example FACS plot of a new phenotypic population isolated during the week 4 sorting process: DN-Late (refer to Fig. S1). During the week 4 sorts, the DN population of the DP cells (7.64% DN population) were additionally sorted. These cells were FACS analyzed along with the other intended cell populations during week 5. (**E**) Barchart displaying cytometry data for puromycin and zeocin selected cell lines detailing percentages of each phenotypic state, performed in duplicates, where cells were initially gated for all fluorescent populations at week 2 (G. R, and DP) which were plated and resorted the following week.

Following two weeks of puromycin selection (2 µg/ml), we categorized cells into one of four distinct expression phenotypes based on their expression of fluorescent markers mRuby3-NLS and mNeonGreen-NLS: Double Negative (DN), Green only (G), Red only (R), and Double Positive (DP; Fig. 1B). While these show intracolony homogeneous expression, we also observed colonies with intracolony heterogeneity at the end of week 2, suggesting that silencing occurs post integration. We further performed FACS analysis which showed the vast majority of cells existed in the DN state, while less than 1% of cells expressed our desired DP phenotype even after cells had been subjected to antibiotic selection (Fig. 2A). High-transfection efficiency is observed in HEK293T, and these cells initially exist in the double positive state (> 99% from days 1–4, data not shown). However, as transient expression decreases from days 4–14, we see the emergence of the four distinct expression phenotypes shown above. Next, we set out to interrogate potential sources of this expression heterogeneity.

Expression phenotype silencing dynamics are controlled by screening and selection pressure.

After sorting cells into the four distinct expression phenotypes, we set out to characterize how silencing dynamics are affected by screening pressure and selective markers. After performing a sorting step at the end of week 2 post-transfection, using fluorescent gates of 10^4^ au, biological duplicates of each expression phenotype were cultured for an additional 3 weeks and sorted once a week (Fig. 2A). For example, the DP cells were sorted again at the end of week 3 and only the DP population from that sample was continued in culture. Crucially, the fraction of DP cells that transition into the DN state at week 3 would represent the fraction silenced per week. We refer to a cell transition as the temporal changes in the fraction of cells in each state from one sort to the next. Figure 2A shows representative plots from one biological replicate for the DP state at week 3, 4 and 5. We see the proportion of cells that are silenced (transition from the DP state to the DN state) decreases from 21.37% in week 3, to 7.64% in week 4 and 3.12% in week 5.

The silencing dynamics for the other expression phenotypes are summarized in Fig. 2B. We see that while the DN state is very stable (no visible reversion to a fluorescent state), the G and R state follow similar dynamics to the DP, although from different initial conditions. Refer to Fig. S1 for weekly sorts from each replicate for each expression phenotype. Additionally, the rate of change follows a first order trajectory. Thus assuming a first order reaction rate, we calculated the silencing rate constant or “half-life of the active state” by taking the ratio of new cells silenced from week to week (Fig. 2C). For the DP cells, this constant is 2.2 ± 0.1 per week. Additionally, the same asymptotic trend is shown in Fig. 2B between all three fluorescent expression phenotypes, along with similar silencing rate constants in Fig. 2C.

We also measured the reversibility of our model system during this time frame. DN-Early cells, initially sorted at week 2, demonstrated a return to the fluorescent states of only 0.23% cells after one week (week 3; Fig. 2B) and had less than 0.02% fluorescing cells at week 5. We also sub-cultured DN cells taken from the week 4 DP sort that had transitioned into the DN state, termed DN-late. These DN-Late cells showed around 2% fluorescing cells during the following sorting session (week 5; Fig. 2D) exhibiting increased reversibility of silencing.

Additionally, to see if the use of selective markers (without screening pressure) can also affect our model system, we used the antibiotic selection markers that flank our fluorescent reporters separately to impart selective pressure onto our model system. Figure 2E shows that when using puromycin to select, the Green only expression phenotype dominates, while when using zeocin to select, the Red only expression phenotype dominates. This effect has not previously been observed in modular systems.

Expression phenotypes are controlled by epigenetic factors.

Based on this work, we might expect multiple epigenetic states to be active in our model system including methylation and histone modifications. However, to definitively rule out the silencing of individual TUs on the basis of any sequence alterations as a result of integration errors, we examined sorted fluorescent and non-fluorescent cell lines for the presence of the fluorescent marker sequences. Genomic PCRs showed the presence of fluorescence marker sequences in the non-fluorescent cells (Fig. S2). Additionally, using RNA-FISH, we found that fluorescent marker RNA was absent in the silenced cells, while being present within the fluorescent cells in the same field of view (Fig. S2). These assays were performed on a 3-TU construct shown in Fig. S2A.

These results suggest that while the correct DNA sequence is present, any subsequent silencing may be occurring through epigenetic mechanisms. To conclusively demonstrate that silencing is epigenetically caused, we used small-molecule inhibitors of these epigenetic processes to partially reverse the silencing of expression phenotype in DN cells both in the early and late phase samples. We used two small molecule inhibitors involved in these pathways: 5-AZA-2′-deoxycytidine (5-Aza-dc) and Trichostatin A (TSA). 5-Aza-dc integrates in place of cytidine within newly formed DNA strands and forms covalent bonds with DNMT1 when the enzyme attempts to methylate it^24^. DNMT1 proteins, which become trapped on DNA, can no longer function and eventually are degraded as part of the DNA damage repair response. This downregulation of DNMT1 activity results in genome wide hypomethylation and the reactivation of previously silenced genes which now remain unmethylated. TSA acts as a selective inhibitor of Histone Deacetylase (HDAC) proteins which normally function to promote chromatin wrapping by removing acetyl groups on histones^25^. By inhibiting HDAC proteins, acetyl groups will persist, DNA will not be bound, and chromatin should shift towards a more open and accessible state allowing for increased expression.

**Figure 3:**
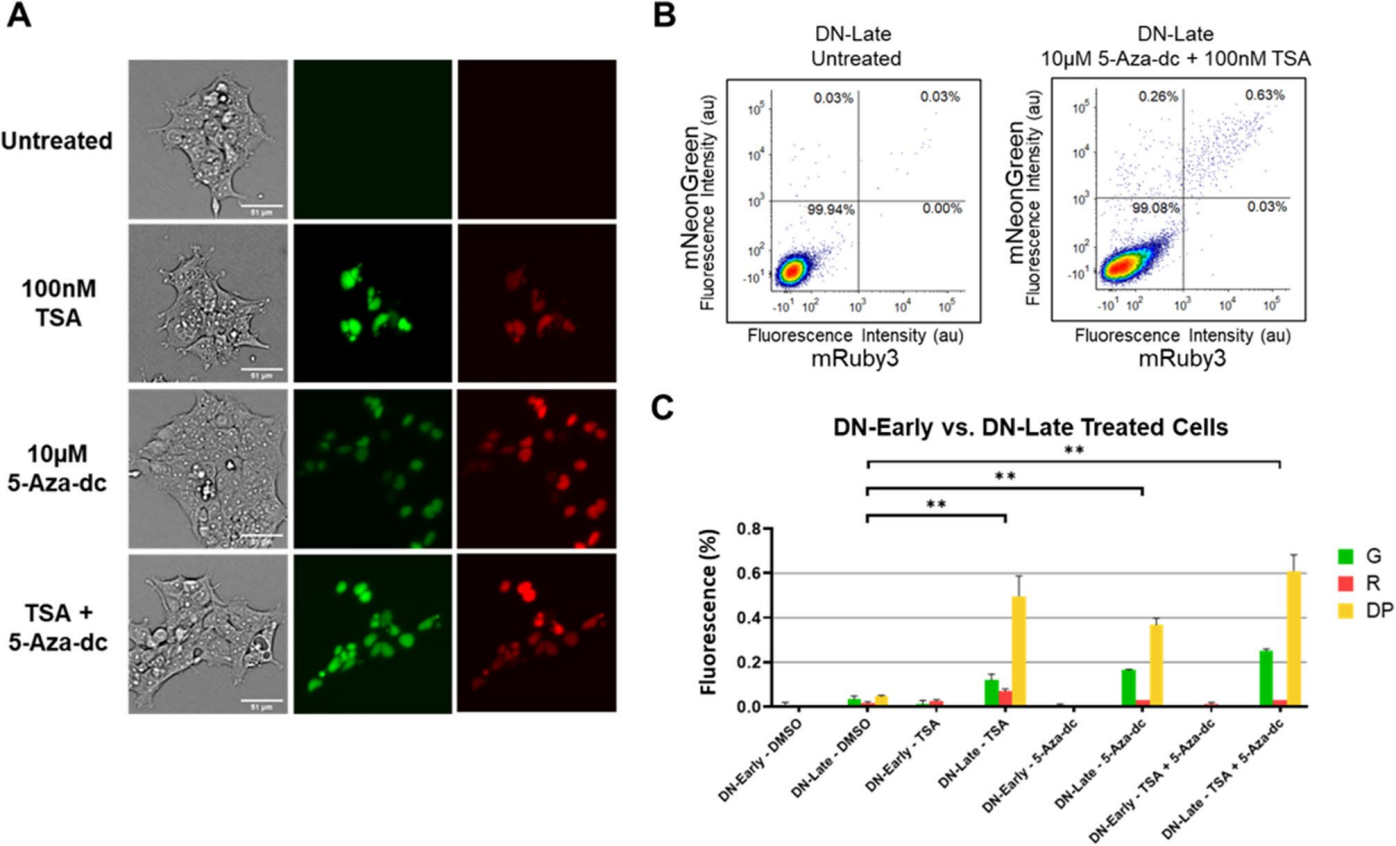
Reversibility of the epigenetically silenced state via epigenetic intervention through small molecule inhibition. (**A**) Representative microscopy images of treated DN-Late cells with either 10 µM 5-Aza-dc, 100 nM TSA, or a combination of both molecules at similar concentrations. (**B**) Flow cytometry comparing the expression of mNeonGreen-NLS and mRuby3-NLS within untreated DN-Late cells to DN-Late cells treated over the duration of 72hrs with a combination of 5-Aza-dc and TSA. (**C**) Summary of treatment conditions comparing the response of both DN-Early and DN-Late cells to a variety of inhibitory conditions using either Dimethyl Sulfoxide (DMSO), 5-Aza-dc only, TSA only, or a combination of both 5-Aza-dc and TSA. All conditions were performed in triplicate with significant changes (**p < 0.01, Bonferroni-corrected multiple t-Test) represented above.

Early and late phase DN cells were incubated with either DMSO, 10 µM 5-Aza-dc, 100 nM TSA, or a combination of 10 µM 5-Aza-dc and 100 nM TSA. 5-Aza-dc was added fresh every 24 h due to its short half-life^26^, while TSA was added only in the last 24 h due to its high toxicity^27^. These inhibitor concentrations are similar to those used previously in literature^22,27^. We attempted to culture cells continuously in the presence of both inhibitors; however, we found that doing so resulted in drastic loss of viability and the inability to collect enough cells for FACS analysis over the 72 h timeline. Cells were analyzed by flow cytometry after 72 h of being cultured with these inhibitors. Using fluorescent microscopy in Fig. 3A, we show that DN-Late cells form individual fluorescent colonies upon treatment with either small molecule inhibitor. These results were quantified by flow cytometry in triplicate (Fig. 3B and Fig. S3). Figure 3B shows that both TSA and 5-Aza-dc can partially reverse expression phenotypes in approximately 1% of cells, indicating that both histone deacetylation and methylation are causal mechanisms to the silencing of multi-TU transgenes. We found that only late phase cells exhibit significant phenotype reversal into the DP state (p < 0.01 for all treatments in comparison to DMSO control) for all treatment regimes, while early phase silenced cells were only reversed into either G or R states at extremely low frequency (less than 1 in a 1000 cells) (Fig. 3C). Interestingly, the principal reversible transition in the late phase is the DN → DP transition (Fig. 3B), suggesting that late silencing tends to silence more than one TU. The observed differences between early and late silenced cells suggest that changes in local DNA structure at the scale of a single TU (~ 4 kb) can be affected by epigenetic silencing motifs.

ATAC-seq shows expression phenotypes correlate with epigenotype.

After confirming epigenetic silencing of our modular circuits, we wanted to assess how epigenetic motifs constrain individual TUs. To assess this, we profiled chromatin accessibility on each expression phenotype using ATAC-seq. Generally, genome-wide epigenetic profiling methods, such as bisulfite-sequencing, are inherently difficult to apply to modular vectors used in mammalian synthetic biology, such as our model system, due to the presence of repetitive elements (promoters, chromatin insulators, UNS barcodes, etc.). Hence, any next generation sequencing-based analysis is restricted to assessing the gene bodies within each TU and not the promoters that are typically epigenetically modified^28,29^. We chose ATAC-seq as it evaluates chromatin accessibility (the functional outcome of multiple epigenetic mechanisms) and not specific epigenetic mechanisms (such as methylation or histone modifications).

**Figure 4:**
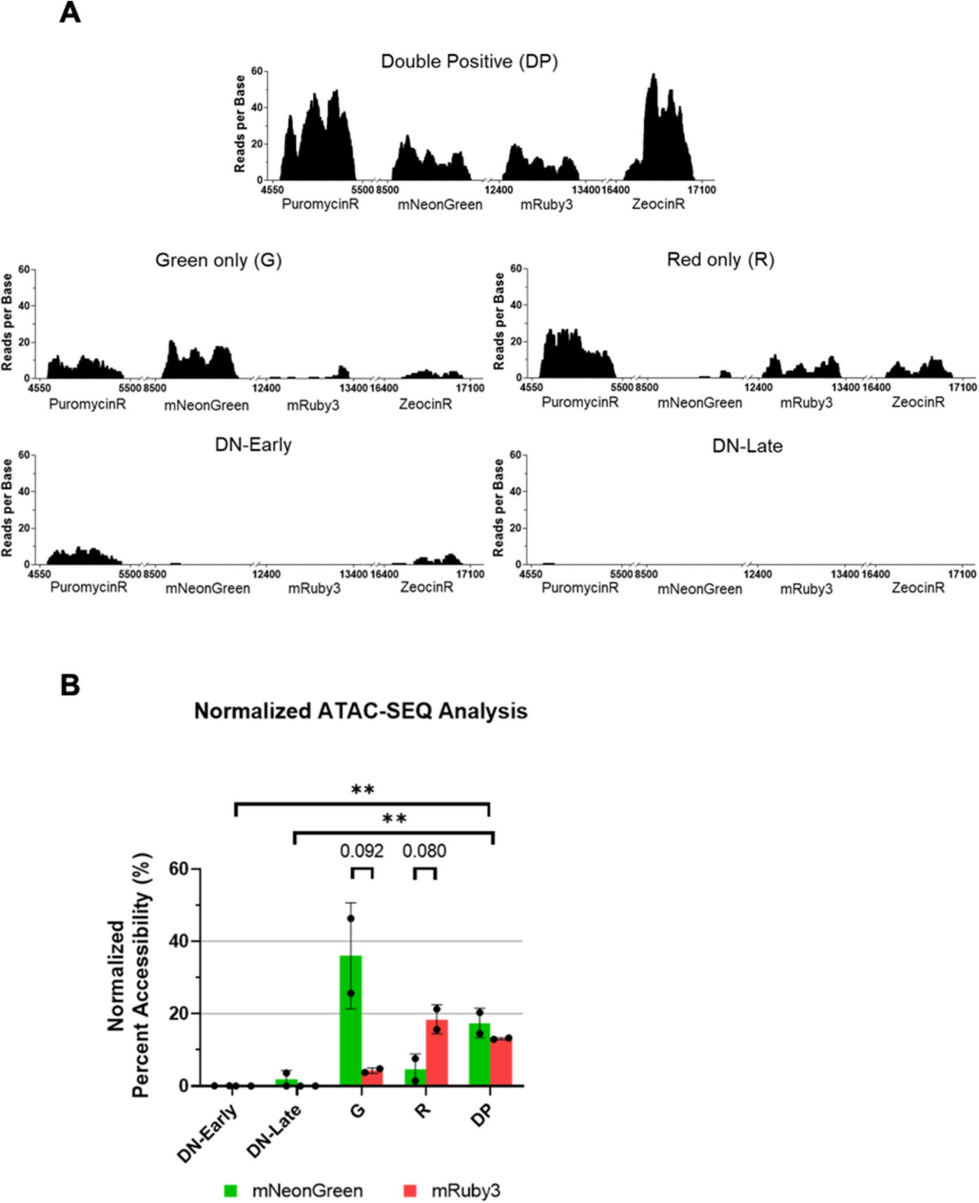
ATAC-seq analysis of genomic DNA derived from sorted and enriched phenotypic populations. (**A**) Representative images displaying ATAC-seq reads associated with each of the constructs’ gene bodies (PuromycinR, mNeonGreen, mRuby3, and ZeocinR) generated for each of the distinct phenotypic states. (**B**) Comparison of fold change as it relates to chromatin accessibility looking at mNeonGreen and mRuby3 between samples. Performed in duplicate with significant changes (**p < 0.01, Bonferroni-corrected multiple t-Test) represented above.

ATAC-seq was performed on our five expression phenotypes generated through our long-term stabilization study (refer to Fig. 2). Reads generated through ATAC-seq which did not align to the human genome were instead aligned to our plasmid as a template. We then filtered for reads only present within the four gene bodies in our model system (PuromycinR, mNeonGreen, mRuby3, ZeocinR) for each sample (replicates shown in Fig. S4 and procedure explained in Fig. S5). We specifically excluded repetitive regions in our construct such as terminators, insulators and promoters due to the inability to ascertain which gene body a short NGS read would be associated with. Representative replicates for each expression phenotype are shown in Fig. 4A (also refer to Fig. S3). In Fig. 4A, we see there are observable differences in gene body accessibility (measure by reads per base) between different expression phenotypes. In Fig. 4A we see the presence of some mRuby3 reads in the Green only phenotype and the presence of some mNeonGreen reads in the Red only sample. This further reinforces the while the fluorescent phenotype is absent the genotype is present suggesting an underlying epigenetic mechanism is causing the silencing.

To allow for statistical comparisons, ATAC-seq data needs to be normalized. Normalization of reads within samples was performed by comparing reads with the gene bodies to plasmid segments in the flanking regions that were equally accessible in all samples (Fig. S6), no differences were observed when data was normalized to endogenous loci (data not shown). After normalization, the percentage accessibility is calculated, enabling comparison between samples. Figure 4B shows that ATAC-seq accessibility, or epigenotype, matches the expression phenotype, as expected. When comparing accessibility within a sample, both the G and R expression phenotypes show decreases in accessibility for the non-fluorescing marker, though they are not statistically significant (p = 0.092 and 0.080 respectively). Comparisons of fluorescent marker accessibility across samples also show statistically significant changes in accessibility (p < 0.006). For example, both fluorescent markers have significant decrease in accessibility between the DP and DN-early phenotypes (Fig. 4B).

The only significant difference between the DN-early and DN-late samples is in the selection marker accessibility (p < 0.016, refer to Fig. 4A and Fig. S7). This shows that the DN-late phenotype has all gene bodies decrease in accessibility, while the DN-early phenotype only has decrease in the fluorescent phenotypes. This reinforces the previous observation that early silencing has short range implications (single TU), while late silencing targets the entire integrated construct.

## Discussion

Herein, we used a model multi-gene system to understand expression heterogeneity in integrated multi-gene genetic circuits. Our data suggests this differential expression phenotypes can in part can be explained by epigenetic silencing after integration. This demonstrates the need to understand the resulting localized genomic environment that arises after the integration of multi-gene transcripts.

The dramatic changes in expression heterogeneity emerged during the second week of culture after transfection. We associated these transient expression changes with the degradation of transient plasmids, any remaining mRNA, and proteins in the second week after transfection, as the phenotypic state of each cell was actualized. Then by applying constant screening pressure (one sort per week), we observed changes in silencing dynamics over time. The expression of selected fluorescent states appears to stabilize in our model system over the course of a month through a constant decrease in the proportion of newly silenced states with each sort. Given that puromycin selection occurred for two weeks and that all expression phenotypes were stably integrated and fluorescent when sorted, we expect that any subsequent silencing is likely not caused by sequence alterations due to integration. Hence, this suggests other silencing mechanisms are active from week 2 to week 5. We tried to perform clonal analysis on these samples however, due to instability of these expression phenotypes we weren’t able to culture enough cells to assess these on a clonal level. The lack of clonal analysis means it is not completely possible to rule out other alternative explanations such as non-allelic homologous recombination or genetic drift. Further studies using long-read sequencing on the entire integrated construct will resolve this.

The plasticity (ability to change phenotypic state) of the DN-late cells when compared to the DN-early cells suggests that the permanence of specific epigenetic states might be linked to the time from the establishment of these states as well as the time since integration of the genetic constructs. Previously, different epigenetic silencing states have been shown to have varying reversibility characteristics^30^. In particular, it is known that methylation and histone modifications can dominate different stages of silencing, which we will investigate below. Additionally, selection markers were shown to favor expression phenotypes associated with proximal genes, indicating that selective pressure appears to change the distribution of expression phenotypes. This suggests that both selection and screening pressure can force the local epigenetic environment to remodel over time. As the changes in expression are not occurring at the transcriptional or translational level, but rather, epigenetic modifications at the DNA level and histone level are likely dictating local chromatin conformation and transcription factor accessibility.

This was seen in the expression plasticity observed in DN-late cells and could result from an incomplete stabilization of epigenetic mechanisms, which have not had time to become fully established. Because of this, we were able to intervene with inhibitors of these mechanisms to partially restore fluorescent expression. Meanwhile, DN-early cells had several weeks to properly reinforce these silencing mechanisms wherein no recovery of fluorescence was observed. While our model system employed chromatin insulators upstream of every TU, this was not sufficient to isolate our TU from being silencing. Insulators have previously been shown to play a significant role in regulating the epigenetic silencing of transgenes through the control of local DNA structure and accessibility^15,22,31,32^. Hence, we employed the A2 chromatin insulator^17^ that has previously been shown to have a greater insulating capacity over the canonical cHS4 chromatin insulator^17^. While such CTCF-binding motifs can themselves be methylated^33^, this will necessitate future modifications of chromatin insulators to be resistant to epigenetic modifications.

While integration-mediated recombination is not expected to affect our samples (as transient Cas9 RNP expression is minimal after two weeks) we have not definitely ruled out that the observed silencing effects are not caused by non-allelic homologous recombination (NAHR). We intend to perform long-read sequencing on our samples in the future to assay this mechanism. NAHR occurs between repeated transposable elements (such as the 300 bp Alu sequences)^34^ are inversely correlated to inter-repeat distance^35^. As NAHR is usually studied using endogenous sequences, our modular constructs could provide a unique exogenous model system to study this genetic mechanism.

Subsequent studies of these model systems should target specific epigenetic mechanisms. One example would be using SMRT sequencing (Pacific Biosciences) to assess the DNA methylation status of promoter and chromatin regions in our modular system. We anticipate that by modifying chromatin insulator sequences and design^17,22,28^ or changing their number and orientation^31,36^, we could be able to positively impact the performance of integrated modular genetic circuits. Additionally, we expect the ability to screen for integration using splice acceptor systems^37^, also known as promoter traps^38^, should increase circuit performance by imparting selective pressure on cells where the circuit is integrated and not just being transiently expressed. This work suggests the ability to select or screen for integration from both ends of the modular system will lead to significant decreases in TU silencing and consequent increases in the performance of integrated genetic circuits.

## Conclusion

In this study, we developed a model system to investigate the causes of expression heterogeneity in stably integrated multi-transcript unit genetic circuits. Our model system was used to show that expression heterogeneity is not caused by alteration to the coding sequence during integration but instead results from local changes in chromosomal accessibility leading to epigenetic silencing of individual transcript units. We demonstrated that expression phenotypes are clearly reflected in local changes in chromatin accessibility within the integrated genetic constructs. This work suggests that new mammalian synthetic biology approaches are required to resist epigenetic silencing mechanisms that degrade the performance of modular genetic circuits and further stresses the need for synthetic biology to engineer the epigenome.

## Methods

### Cloning of plasmid constructs

Unique Nucleotide Sequences (UNS) were designed by Casini et al.^39^ and used to assemble various chromatin insulator, promoter, genes, terminators and other genetic elements in a hierarchical assembly using the UNS as overlaps for Gibson assembly similar to previous methods described^23^. All DNA fragments were either chemically synthesized from primers (designed by DNAworks^40^ or PCR-amplified from existing plasmids). All DNA syntheses were performed by Integrated DNA Technologies (Coralville, IA). Plasmids were sequence-verified by Genewiz (San Diego, CA). Annotated plasmids maps for relevant sequences are provided in Figs. 1A and S2A and in Supplementary information.

### Cell culture, transfections, and selection

HEK293T cells obtained from ATCC were cultured in Dulbecco’s modified Eagle’s medium (DMEM; Corning) containing 10% Fetal Bovine Serum (FBS; Seradigm) with 1% Penicillin and Streptomycin (GenClone) and incubated at 37 °C and 5% CO_2_. 20,000 cells were seeded into each well of a 24 well plate 24 h before being transfected. Cells were transfected in duplicates as per Promega Fugene HD manufacturer’s protocol. Each transfection was performed using 2 µg of DNA consisting of 1 µg of our multi-gene construct plasmid (pJZ1506), 0.33 µg of Cas9 plasmid (pJZ0387), and 0.66 µg of gRNA plasmid (pJZ0338). Within various experiments, cells were either selected with puromycin (Invivogen) at a concentration of 2 µg/mL or zeocin (Alfa Aesar) at a concentration of 100 µg/mL. Cells were gently washed with fresh media containing antibiotics every two days over the course of two weeks (PBS was not used as we experienced noticeable cell detachment).

### Genomic PCR

Genomic DNA was extracted using the Quick-DNA Midiprep Plus Kit (Zymo Research) according to manufacturer's instructions immediately after FACS sorting. PCR was done using primers that contained the first unique sequence on each gene on the 5′ end and the last unique sequence on the 3′ end that had an annealing temperature of near 60 °C For the mNeonGreen gene, the forward and reverse primers were 5′-AGAGGACAACATGGCC-3′ and 5′-CTTGTACAGCTCGTCCA-3, respectively.

### FACS sorting and analysis

Sorting was performed through the UCI Institute for Immunology Flow Core Facility using the BD FACSAria Fusion Sorter with a 70 µm nozzle. For the first sort at two weeks, we collected as many cells as we could, capped at 50,000 cells, for each of the phenotypic states. In the following weeks 50,000 cells were collected for each of the four phenotypic states, Double Negative (DN), Green only (G), Red only (R), and Double Positive (DP), based on their expression of mNeonGreen and mRuby3 gated at 10^4^ au. All four distinct populations were cultured separately and resorted every 7 days for their respective phenotypes within all samples (Ex: GFP only expressing cells were sorted and replated within the G only samples each week). Cells treated with zeocin were screened for all four distinct populations, but only fluorescent cells (DP, G, and R) were collected and cultured together each week. Data was analyzed using FCS Express 7.

### Flow cytometry and analysis

Flow Cytometry was performed through the UCI Stem Cell Research Center Flow Cytometry Core using the BD Fortessa. 200,000 cells were processed for each of the samples, and data was analyzed using FCS Express 7. Plots were designed displaying 50,000 events each and reflect identical fluorescent compensation values.

### ATAC-sequencing (assay for transposase-accessible chromatin using sequencing) preparation

Cells were collected the next day following our 5-week FACS sort timepoint in which 50,000 live cells were prepared for ATAC-sequencing following the Corces et al. Omni-ATAC protocol^41^. Briefly, the cells were washed with PBS and counted to isolate 50,000 live cells. The nuclei were isolated and then pelleted by centrifugation at 500×*g* for 5 min at 4 °C. The DNA was transposed by the UCI Genomic High-Throughput Core Facility. The DNA was then purified using MinElute PCR Purification Kit (Qiagen), and the UCI Genomic High-Throughput Core Facility performed the final ATAC-seq library preparations and sequencing. ATAC-seq libraries were sequenced on 1 lane of a NovaSeq 50 bp paired-end run to an average 30 million reads.

### ATAC-seq data analysis

Raw reads from the 10 samples were first QCed (fastqQC, v0.11.7) and trimmed using trimmomatic (v0.35). Trimmed reads were then aligned to the hg19 build of the human genome using Bowtie2 (v2.2.7) with alignment parameters: bowtie2-X 2000-dovetail. Potential PCR duplicate reads were marked using MarkDuplicates from the Picard tools (v1.130). Black list regions in the human genome (Stanford version) were excluded and peaks were called using MACS2. Unmapped reads were then extracted from the alignment and further aligned to the multi-TU plasmid sequence and reads aligned to the four regions of interest were processed and counted using samtools (v1.9) and featureCounts from subread (v1.5.0).

### Statistical methods

A minimum of two independent experiments were carried out (FACS-based phenotype characterization and ATAC-seq), and where possible three biological replicates were assayed. Values represent average ± SEM and were subject to the Bonferroni-corrected two-tailed Student’s t test. Values of *p < 0.05, **p < 0.01, and ***p < 0.001 were considered statistically significant.

### Small molecule inhibition

Previously transfected HEK293T cells that were FACS sorted into the phenotypic population of Late-DN and Early-DN were seeded at 40,000 cells on a 6 well plate. Cells were treated with either 5-Aza-dc (SelleckChem) at 10 µM, TSA (Apexbio Technology) at 100 nM, or a combination of both at the same concentrations. Cells treated with either 5-Aza-dc only or TSA only were treated for 72 h with a media change and reintroduction of the respective inhibitor every 24 h. When used in combination, cells were treated with 10 µM 5-Aza-dc for 72 h and 100 nM TSA was additionally added for the last 24 h. Media was changed every 24 h similar to the single inhibitor treated wells. Experiment was run in triplicates.

## Supplementary Information

Supplementary Information.

## Abbreviations

PCR: Polymerase chain reaction
TU: Transcription unit
HEK293T: Human embryonic kidney cell line 293 T
DN-late: Double negative “late”
DN-Early: Double negative “early”
DP: Double positive
G: GFP only
R: RFP only
DNMT1: DNA methyltransferase 1
5-Aza-dc: 5-AZA-2′-deoxycytidine
TSA: Trichostatin A
ATAC-seq: Assay for transposase-accessible chromatin using sequencing
CTCF: CCCTC-binding factor
