# Supplementary material for: Epigenetic silencing directs expression heterogeneity of stably integrated multi-transcript unit genetic circuits
Source: Sci Rep. 2021 Jan 28;11:2424. doi: 10.1038/s41598-021-81975-1 (PMC7844226; doi:10.1038/s41598-021-81975-1)
Supplement: Supplementary file 1 — Supplementary Information. [file 41598_2021_81975_MOESM1_ESM.pdf]

Epigenetic silencing directs expression heterogeneity of stably integrated multi-transcript unit genetic circuits.

SUPPLEMENTARY INFORMATION

Jan Zimak* [a,b], Zachary W. Wagoner* [a,b], Nellie Nelson [a,b], Brooke Waechtler [a,b], Hana Schlosser [a,b], Morgan Kopecky [a,b], Jie Wu [c,f], Weian Zhao [a,b,c,d,e,f]

*Contributed equally to this project

a Sue and Bill Gross Stem Cell Research Center, University of California, Irvine, Irvine, CA 92697, USA
b Department of Pharmaceutical Sciences, University of California, Irvine, Irvine, CA 92697, USA
c Chao Family Comprehensive Cancer Center, University of California, Irvine, Irvine, CA 92697, USA
d Edwards Life Sciences Center for Advanced Cardiovascular Technology, University of California, Irvine, Irvine, CA 92697, USA
e Department of Biomedical Engineering, University of California, Irvine, Irvine, CA 92697, USA
f Department of Biological Chemistry, University of California, Irvine, Irvine, CA 92697, USA

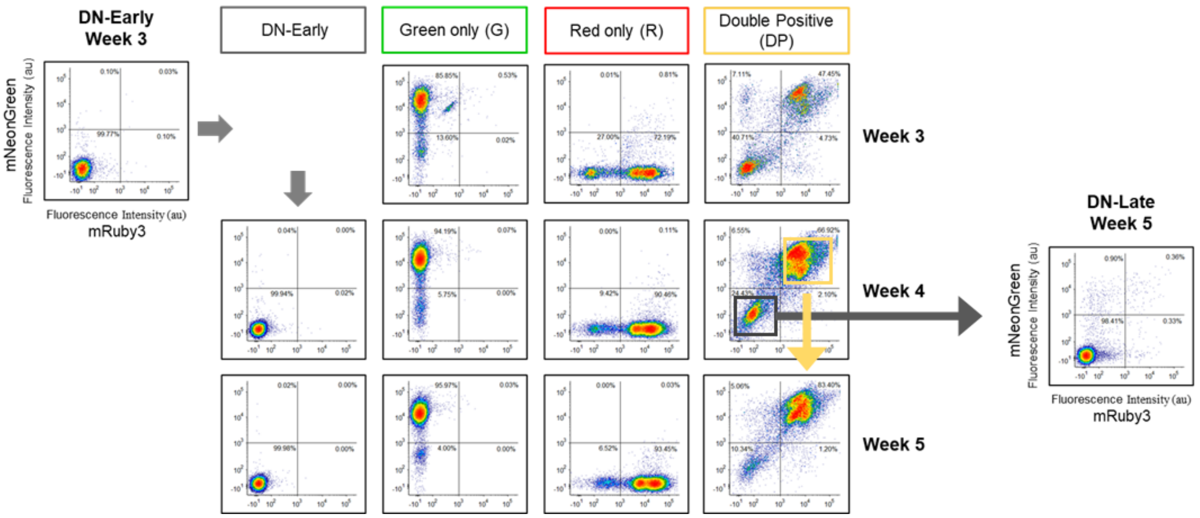

Supplementary Figure 1. Replicate FACS plots for weeks 3 through 5 as part of the 5-week characterization study, shown in Figure 2. Plots display the expression of mNeonGreen (y-axis) and mRuby3 (x-axis) where each week cells were sorted for the fraction of cells of each population phenotype (i.e. fraction expressing both mNeonGreen and mRuby3 (47.45%) of week 3 DP population were sorted and cultured for FACS the following week). A new population was generated during the week 4 sorts deemed "DN-Late" in which the non-fluorescent expressing fraction of cells in the week 4 DP population were sorted out and cultured for analysis during the final week 5 sorts.

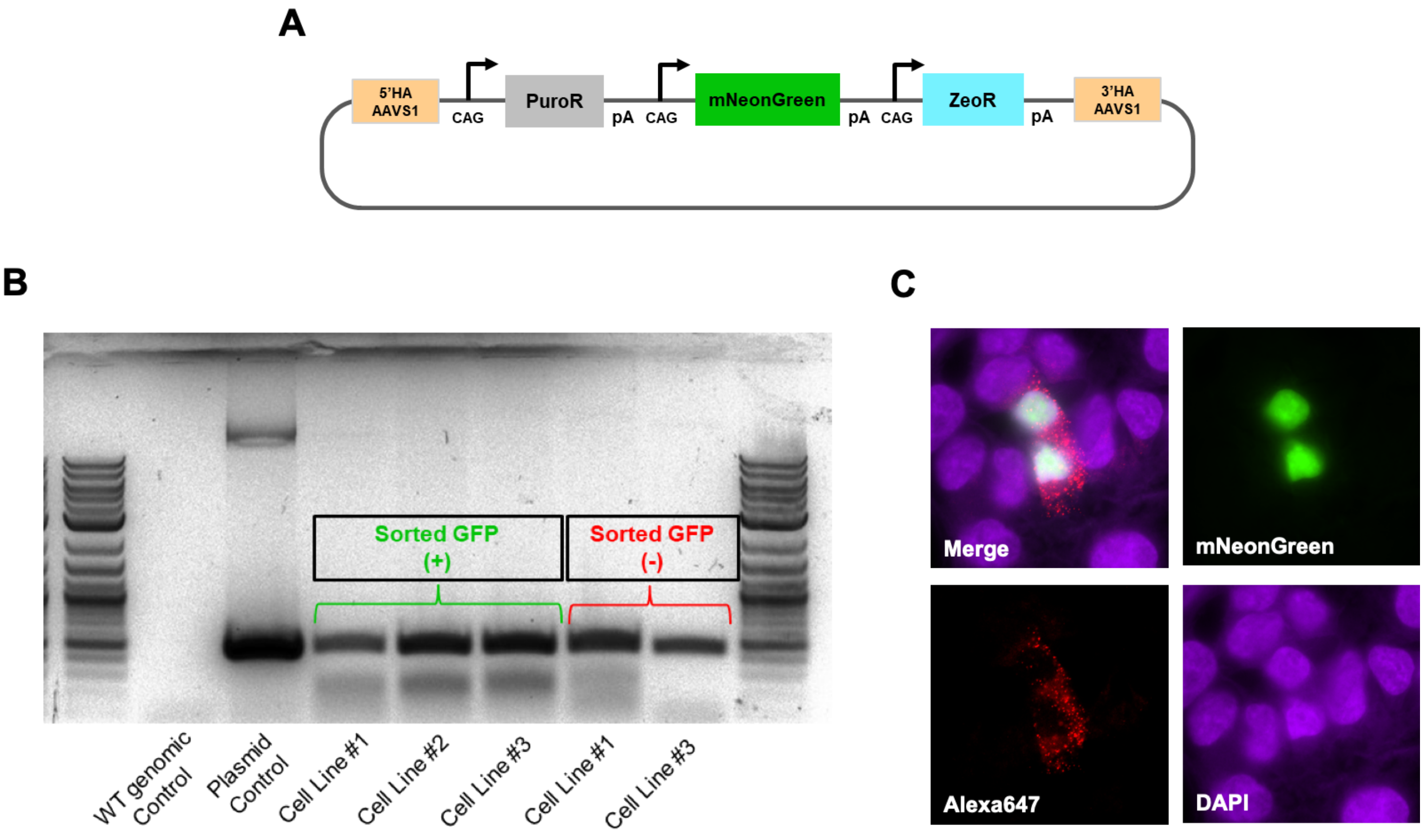

Supplementary Figure 2. (A) Simple schematic showing a multi-gene construct designed to express three genes (Puromycin Resistance (PuroR), mNeonGreen-NLS (GFP), Zeocin Resistance (ZeoR)). Expression of all genes are driven by independent constitutive CAG promoters, followed by WPRE-bGHpolyA (pA - not shown) sequences. The multi-gene transcripts are then flanked by 5' and 3' homology arms (HA) directing insertion into the AAVS1 loci. (B) Several cell lines were transfected with our construct and FACS sorted at 14 days, following several rounds of puromycin selection (2µg/ml), into GFP (+) and GFP (-) populations. PCR amplification was performed probing for the entire mNeonGreen gene within three GFP (+) and two GFP (-) cells lines purified during the previous FACS step. DNA extracted from non-transfected HEK293T cells and our multigene construct acted as negative and positive controls, respectively. (C) RNA-FISH was performed on an engineered GFP expressing cell line, which exhibited heterogeneous expression of our construct, probing for mNeonGreen RNA. The cell line was generated by transfecting HEK293T cells with our construct and selecting with puromycin every two days for a total of 14 days with all cells demonstrating a resistance to puromycin by the end. No FACS Sorting was performed allowing us to easily compare naturally silenced cells to fluorescing cells within a single colony.

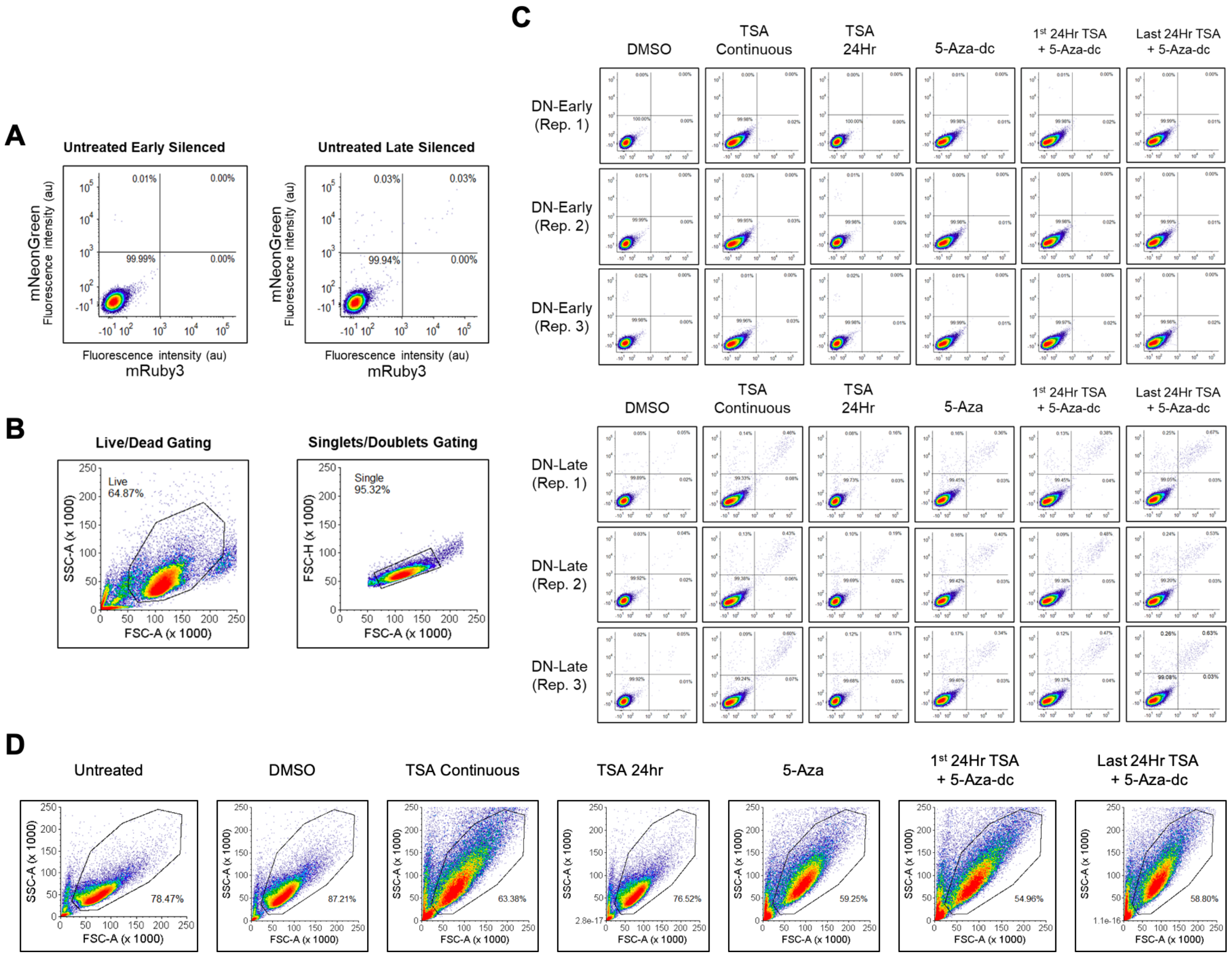

Supplementary Figure 3. Flow cytometry analysis of DN-Early and DN-Late cells with epigenetic inhibitors. (A) Untreated DN-Early and DN-Late cells run through FACS. (B) Representative images of gating process for "Live" parent population and "singlet" sub-population from which final FACS plots comparing the expression of mNeonGreen (Y-axis) and mRuby3 (X-axis) were generated. (C) Replicate FACS plots for inhibitor treated DN-Early and DN-late cells not visualized in the publication main figure (Figure 3B/C). (D) FSC/SSC plots and gating showing toxic effects from different inhibitor treatments showing gating to exclude toxic effects from the inhibitor toxicity and gating from each inhibitor condition

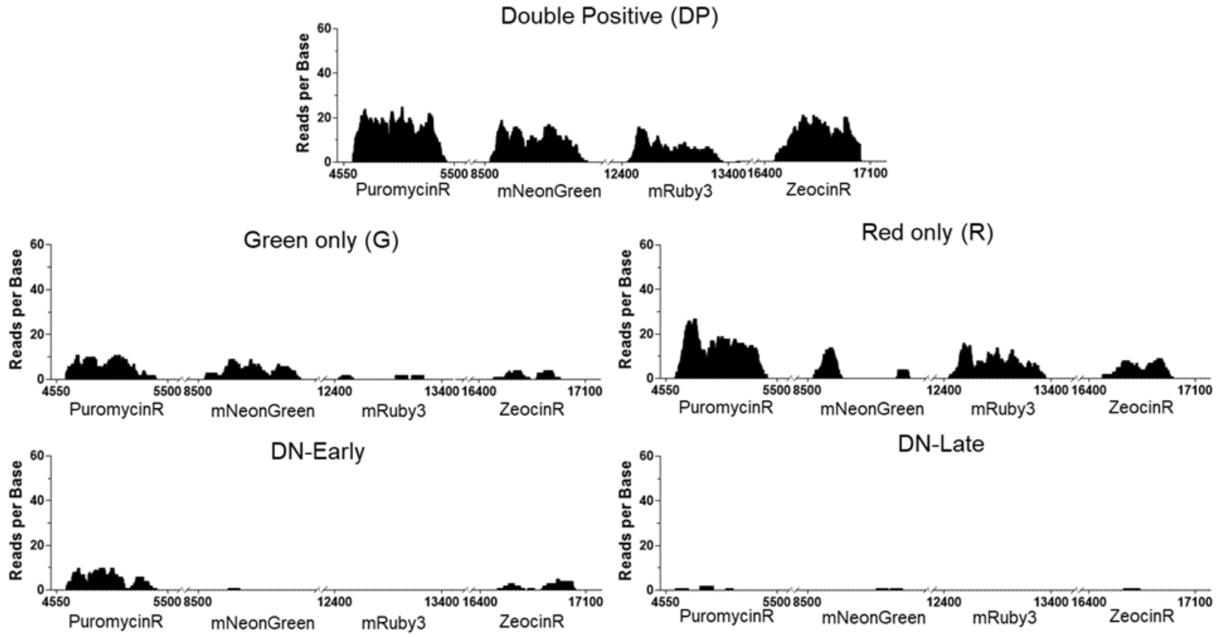

Supplementary Figure 4. Additional ATAC-seq replicates not shown as representative images in Figure 4 displaying the number of reads acquired within the four gene bodies of our construct (PuromycinR, mNeonGreen, mRuby3, and ZeocinR).

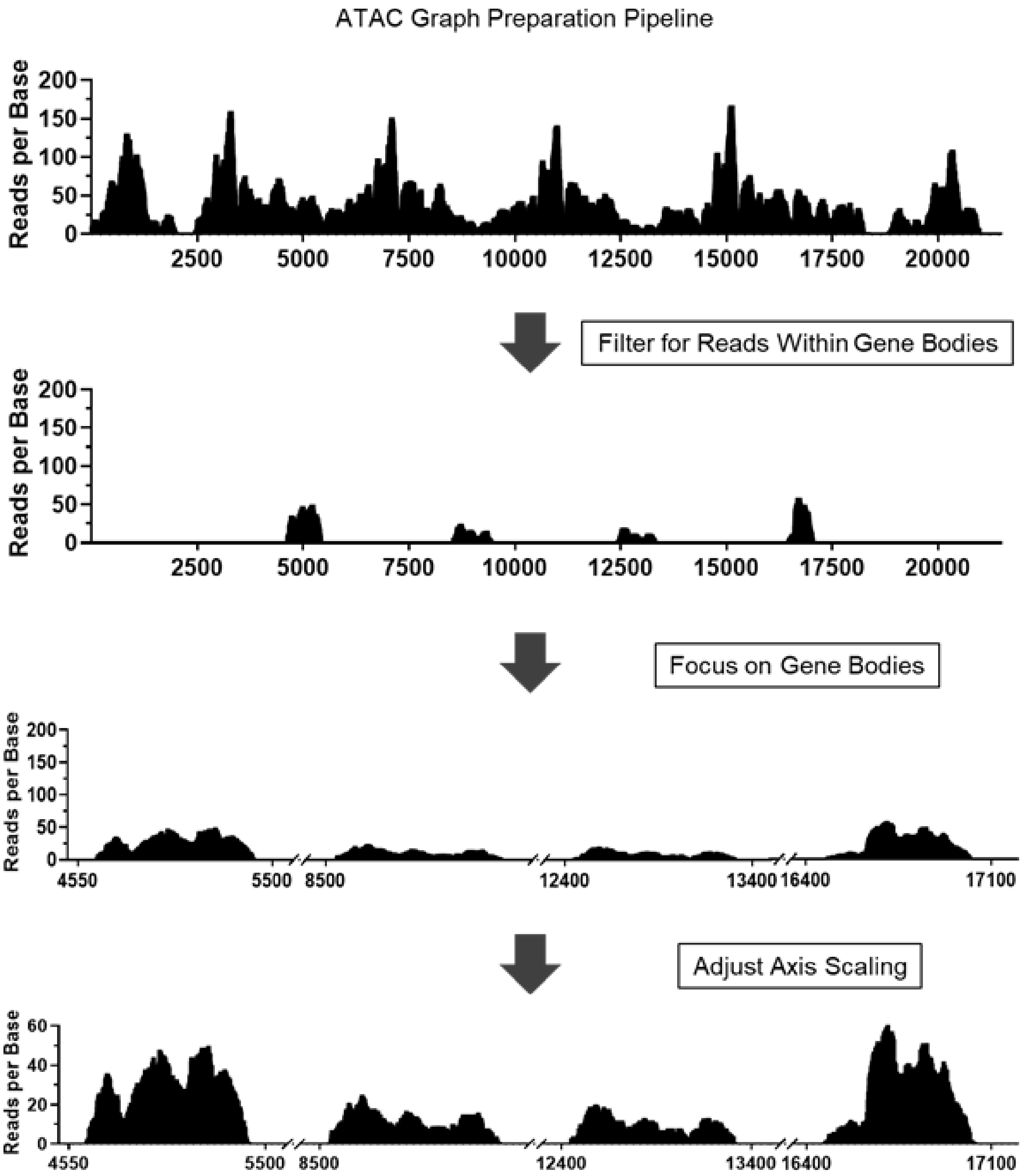

Supplementary Figure 5. Pipeline used for processing ATAC-seq reads. Reads were acquired from the UCI Genomic High-Throughput Sequencing Core and aligned to our plasmid construct as a template. These reads were then filtered to pull out all reads which aligned only to the gene bodies within each gene. From there, the x-axis was adjusted to focus on those reads with the last step being readjusting the y-axis to accommodate for the lower number of reads present in the gene bodies as opposed to other higher read regions (CAG sequences). Double Positive ATAC-seq sample was used in this example.

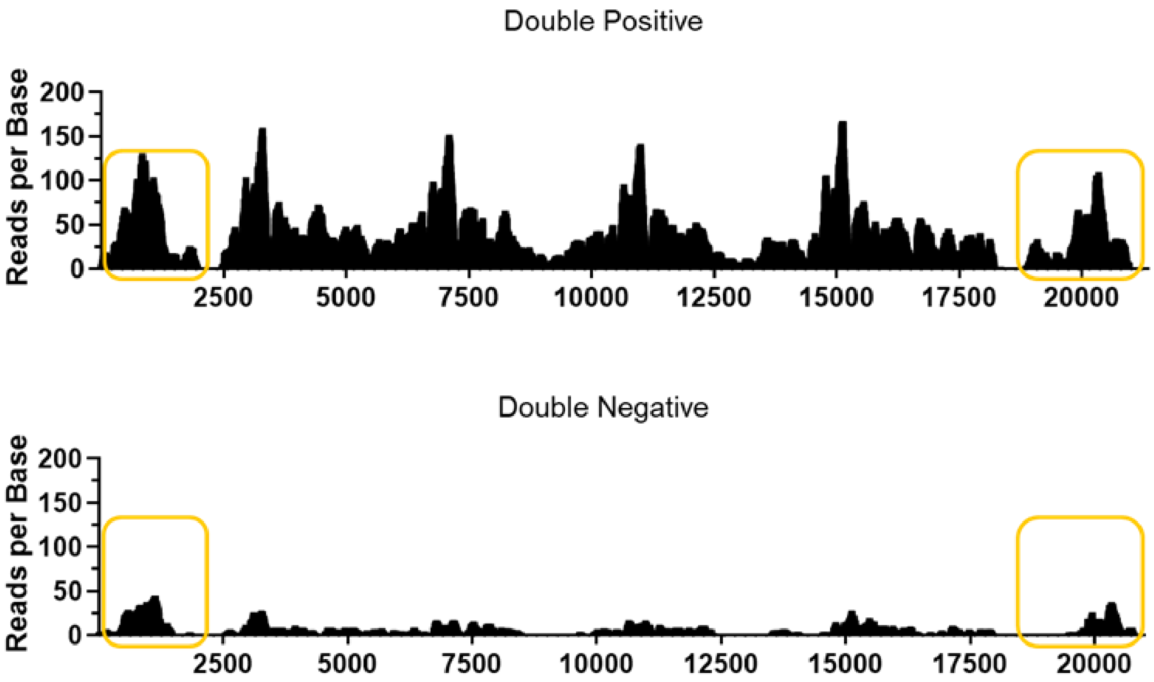

Supplementary Figure 6. Reads from flanking plasmid regions were used to normalize reads within the gene bodies presented in the ATAC-seq data (shown in orange boxes for the sorted reads from two samples)

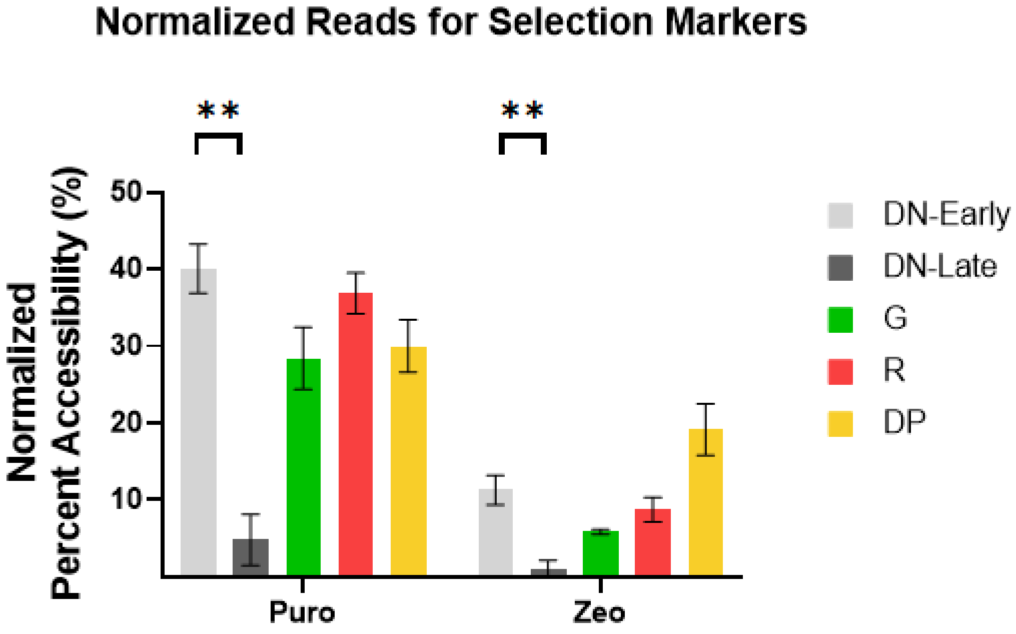

Supplementary Figure 7. Normalized ATAC-Seq data for selection marker accessibility
